# Psychological Safety in Surgical Training

**DOI:** 10.7759/cureus.103862

**Published:** 2026-02-18

**Authors:** Xiang Yuen Po

**Affiliations:** 1 General Surgery, Royal Melbourne Hospital, Melbourne, AUS

**Keywords:** clinical learning environment, future surgeon, psychological safety, surgical education, surgical training programme

## Abstract

Psychological safety is the belief that individuals may ask questions, express doubts, and admit mistakes without fear of humiliation. It has emerged as a fundamental determinant of learning in high-stakes clinical environments. Surgical education remains historically hierarchical, often privileging endurance over vulnerability and silence over inquiry. While operative exposure and technical skill acquisition remain central to training, emerging evidence demonstrates that hierarchical and punitive workplace cultures impair error disclosure, suppress speaking-up behaviours, reduce feedback uptake, and increase cognitive load during performance, thereby limiting skill consolidation and patient safety improvement. This paper adopts a narrative review and conceptual synthesis approach, drawing on contemporary surgical, organisational, and educational literature to examine the mechanisms, barriers, and practical applications of psychological safety within surgical training. This paper argues that psychological safety is not merely a cultural aspiration but also a critical determinant of effective surgical training. The author reviews historical and contemporary models of surgical teaching, examines barriers, including hierarchy and punitive error responses, and proposes practical strategies for cultivating safe learning environments. The surgeon of the future must be developed not only through procedural volume but through mentorship that supports curiosity, reflection, and compassion. A psychologically safe surgical culture produces faster learners, safer decision-makers, and more ethically grounded clinicians.

## Editorial

Introduction

A surgical registrar stands at the operating table during a complex case. The anatomy is unclear, and bleeding obscures the field. The consultant appears pressed for time. The registrar hesitates, uncertain whether to clarify the dissection plane or proceed. In that moment, the decision to speak or remain silent may shape not only patient outcomes but also the registrar’s development as a surgeon. Such moments are not uncommon in operating theatres, where hierarchy, urgency, and evaluation converge. They reveal a central but often unexamined question in surgical education: under what conditions do trainees feel safe enough to learn?

Surgical education has traditionally been defined by apprenticeship, repetition, and progressive entrustment of responsibility. While this model has produced clinically capable surgeons for generations, it evolved alongside a workplace culture characterised by steep hierarchy, emotional resilience, and expectations of compliance [1]. With limits on working hours, increasing procedural complexity, and institutional commitments to patient safety, the assumption that exposure alone produces competence is no longer sustainable. Modern educational theory emphasises that learning is relational, reflective, and emotionally modulated, shaped by social interaction, feedback processes, and the learner’s cognitive and affective state [2,3]. When trainees feel unable to ask questions freely or express uncertainty, they default to performance rather than learning behaviour. This phenomenon is amplified in operating theatres, where sociocultural signals are intensified by stress, urgency, and senior authority figures. In this context, psychological safety should be understood not merely as a desirable workplace atmosphere but also as a foundational condition for skill acquisition.

The growing emphasis on patient safety, competency-based training, and professional accountability makes it timely to examine how training culture shapes learning behaviours. As surgical practice becomes more technologically complex and interdisciplinary, the ability to question, reflect, and adapt is increasingly recognised as central to expertise. In this context, psychological safety is not merely a desirable workplace atmosphere but a prerequisite for skill acquisition [4]. This paper explores psychological safety as a primary determinant of learning, synthesises evidence from surgical and non-surgical literature, and offers actionable strategies for embedding safe feedback and cultural transformation within training programs.

Historical context: the silent apprentice

The classical “see one, do one, teach one” paradigm assumes that observation, followed by supervised replication, results in mastery [5]. Historically rooted in the Halstedian apprenticeship model of the late nineteenth and early twentieth centuries, this approach emphasised graded responsibility, immersion in clinical practice, and close mentorship within hierarchical teams. It successfully produced generations of technically capable surgeons and fostered professional identity through longitudinal apprenticeship and operative exposure. Indeed, progressive entrustment and experiential learning remain foundational principles of contemporary surgical training. Missing from the heuristic, however, is explicit recognition that skill development also requires structured error analysis, reflective processing, guided feedback, and iterative refinement.

Traditional surgical culture, while effective in transmitting technical craft knowledge, often constrained these deeper learning mechanisms. Ethnographic and survey studies of surgical training environments have documented patterns of shame-based teaching, public correction, and intimidation as historically normalised pedagogical tools [6,7].

Such norms, including shame-based feedback that increases cognitive load, rigid hierarchy that limits reciprocal dialogue, error concealment that prevents system-level learning, and stoicism in response to intimidation, shaped environments in which resilience was valorised, but vulnerability was suppressed [8-10]. In these settings, survival often equated to success, even if learning efficiency, psychological well-being, and reflective capacity were compromised.

Psychological safety: definitions and mechanisms

Amy Edmondson defines psychological safety as a shared belief that the team is safe for interpersonal risk-taking within a particular context such as a workplace [2]. This concept encompasses the combination of trust, respect, and relational security that permits individuals to ask questions, admit limitations, disclose errors, and challenge decisions without fear of humiliation or retaliation. In the surgical context, such interpersonal risks occur continuously, e.g. when a registrar clarifies anatomy mid-procedure, when a trainee discloses uncertainty about next operative steps, or when a junior team member questions a deviation from standard protocol.

Psychological safety is therefore not merely attitudinal but behavioural: it enables the “learning actions” required for skill acquisition in high-stakes environments. These behaviours include speaking up, seeking feedback, reporting near misses, and engaging in reflective dialogue. When these behaviours are suppressed by hierarchy or fear, learning becomes performative rather than developmental.

Neurocognitive evidence provides a mechanistic explanation for this effect. Acute stress, humiliation, and social threat activate sympathetic arousal and threat circuitry, reducing working memory capacity and impairing executive function [11]. Under such conditions, cognitive bandwidth is diverted toward self-protection and impression management rather than task execution and skill integration. Procedural fluidity declines, fine motor control is compromised, and the likelihood of errors increases. In contrast, when learners perceive relational safety, cognitive resources remain available for deliberate practice, pattern recognition, and real-time error correction.

Beyond cognitive load, psychological safety also influences error detection and adaptive expertise formation [12]. In environments where trainees feel safe to voice uncertainty, early deviations in clinical reasoning are surfaced and corrected before harm occurs. This iterative clarification process strengthens diagnostic accuracy and technical judgment. Furthermore, the ability to apply knowledge flexibly in novel or complex situations depends on reflection, curiosity, and intellectual risk-taking. These higher-order learning behaviours are unlikely to emerge in climates dominated by fear of evaluation.

A brief clinical contrast illustrates this dynamic. In an operating theatre with low psychological safety, a registrar notices unclear bleeding at the edge of the dissection field. The consultant appears impatient. The registrar hesitates, choosing not to clarify anatomy for fear of appearing incompetent. The dissection proceeds, and the bleeding worsens, prolonging the operation. In a psychologically safe theatre, the same registrar states, “I’m not confident about this plane, can we reorient?” The consultant pauses, re-demonstrates the anatomical landmark, and verbalises the decision-making process. The moment becomes instructional rather than punitive. The procedure proceeds efficiently, and the trainee leaves with improved spatial understanding rather than reinforced anxiety. The technical steps may be identical, but the learning yield differs profoundly. In this sense, psychological safety is not peripheral to surgical education; it shapes cognitive performance, behavioural engagement, and long-term professional development at the point of care.

Surgical apprenticeship occurs within a high-stakes, continuously evaluative environment that often elicits psychological defence mechanisms as adaptive responses to perceived threat. Trainees may suppress uncertainty, adopt emotional detachment, overcompensate through maladaptive perfectionism, rationalise adverse events, or avoid feedback in order to preserve professional identity and self-esteem. While these responses can provide short-term psychological protection, their chronic activation may impair reflective learning, reduce error disclosure, and increase vulnerability to anxiety, emotional exhaustion, and burnout. The relationship between psychological pressure and mental health in surgical trainees is cumulative and bidirectional: sustained exposure to hierarchical scrutiny, sleep deprivation, and responsibility for patient outcomes heightens stress responses, and compromised well-being in turn diminishes cognitive performance and adaptive capacity. Professional surgical practice demands exceptional mental readiness, emotional regulation, and resilience. However, resilience should be cultivated through supportive mentorship, graduated responsibility, and reflective practice rather than silent endurance. Consequently, apprentices must proactively safeguard their mental health through adaptive coping strategies, help-seeking, structured reflection, and engagement with institutional supports, recognising that psychological stability is not a sign of fragility but a prerequisite for sustained professional excellence and patient safety.

Evidence that psychological safety improves education

Communication and Reporting Behaviours

Psychological safety strengthens the communication behaviours that underpin learning in surgical environments. Drawing on Edmondson’s framework, psychological safety enables interpersonal risk-taking, including asking questions, admitting uncertainty, and seeking clarification without fear of embarrassment or retribution [2]. In surgical training contexts characterised by steep hierarchy and performance evaluation, low psychological safety predicts avoidance behaviours, silence, and withdrawal, all of which restrict access to corrective feedback and real-time learning opportunities.

Psychological safety is positively associated with speaking-up and event-reporting behaviours, widely regarded as markers of a healthy learning culture [13,14]. Leadership inclusiveness and reduced power distance have been shown to increase psychological safety, which in turn mediates trainees’ intention to report adverse events. In this sense, psychological safety functions not merely as a correlated attribute of positive environments but as a mediating mechanism linking leadership behaviours to reporting actions. Similarly, a stronger safety culture within training programs predicts a greater willingness among trainees to voice concerns in patient-safety scenarios. In operating room settings, qualitative research consistently demonstrates that when hierarchical pressures are mitigated, trainees more readily clarify instructions, question decisions, and engage in feedback dialogue [15]. These communication behaviours are central mechanisms through which teams detect errors early, coordinate effectively, and improve performance.

Trainee Well-Being and Learning Capacity

Psychological safety also exerts measurable effects on trainee well-being, which in turn shapes learning capacity. Studies in surgical training environments have identified supportive, emotionally intelligent, and communicative leadership as key predictors of psychologically safe climates [16,17]. Higher levels of psychological safety are associated with professional fulfilment, engagement, and flourishing, whereas low psychological safety correlates with emotional exhaustion, disengagement, and performance-related anxiety [18].

The relationship between psychological safety and well-being is not merely descriptive. Chronic exposure to environments characterised by evaluative threat and fear of humiliation sustains sympathetic activation and cognitive vigilance, increasing vulnerability to burnout. Burnout, in turn, impairs attention, memory consolidation, empathy, and reflective capacity, which are core components of clinical learning. Psychological safety, therefore, protects not only moment-to-moment educational interactions but also the broader cognitive and emotional resources required for sustained skill development.

Clinical Performance and Patient Safety Outcomes

Beyond communication and well-being, psychological safety influences clinical performance processes relevant to patient safety. By enabling early disclosure of uncertainty and rapid clarification of ambiguities, psychologically safe environments improve error detection and real-time correction. Teams with higher psychological safety are more likely to implement safety protocols consistently, engage in shared problem-solving, and adapt to unexpected intraoperative challenges.

Although direct causal trials linking psychological safety to objective surgical performance metrics remain limited, converging evidence supports a plausible mechanistic pathway: inclusive leadership increases psychological safety; psychological safety increases speaking up and feedback behaviours; and these behaviours enhance coordination, reduce preventable errors, and strengthen adaptive expertise. In this framework, psychological safety operates as a proximal determinant of behaviours that directly affect patient safety and learning outcomes.

Barriers to psychological safety in surgical training

The most pervasive and structurally consequential barrier is entrenched hierarchical authority, in which decision-making power and evaluative control remain concentrated among senior clinicians [19]. Although hierarchy provides clarity of responsibility and accountability in operative settings, steep authority gradients can inhibit trainees from questioning decisions or disclosing uncertainty. This inhibition is amplified in high-stakes environments where time pressure, public scrutiny, and perceived reputational risk heighten fear of negative judgment. Because supervisors simultaneously function as assessors, trainees may conflate learning interactions with performance evaluation, prioritising impression management over intellectual risk-taking. Addressing this structural barrier requires deliberate efforts to flatten authority gradients.

A second barrier is the ongoing presence of shame-based practices, including public correction, sarcasm, or humiliation framed as mechanisms for maintaining standards [20]. While historically normalised and sometimes defended as character-building, such approaches increase cognitive load, impair working memory, and reduce procedural retention under stress [11]. When feedback is experienced as punitive rather than developmental, trainees may focus on avoiding visible error rather than engaging in exploratory reasoning. Over time, this dynamic promotes superficial competence, discourages vulnerability, and fosters error concealment rather than reflective analysis. Transitioning from evaluative confrontation to coaching-oriented feedback represents a necessary cultural shift, and structured faculty development initiatives may help operationalise this change.

Finally, systemic pressures, including workload intensity, fatigue, service demands, and time constraints, create operational conditions that erode psychological safety. High clinical throughput can compress teaching moments and reduce opportunities for structured debriefing, reinforcing a transactional rather than developmental training environment. Burnout among both trainees and supervisors correlates with reduced empathy, diminished teaching quality, and lower tolerance for learner vulnerability. In such contexts, emotional exhaustion becomes normalised, and psychological safety is deprioritised in favour of efficiency. Mitigating these operational pressures requires institutional commitment to protected teaching time, structured debriefing processes, and well-being supports to interrupt the cycle of fatigue and diminished educational quality.

Interventions to cultivate a safe culture

Beyond individual supervisory behaviours, the development of psychologically safe surgical learning environments requires system-level and curricular integration. Formal incorporation of psychological safety principles into departmental policies, trainee orientation programs, and clinical governance structures signals institutional commitment and reduces reliance on individual champions [4,10]. Orientation modules that explicitly define expected communication norms, escalation pathways, and respectful feedback practices show promising early effects on trainee engagement and willingness to seek support during complex cases. However, evidence for long-term behavioural change remains emerging rather than definitive. Additionally, implementation requires protected curricular time, faculty alignment, and administrative coordination, which may be challenging in resource-constrained training programs. Embedding these expectations within accreditation standards and training milestones may strengthen sustainability, but it depends on institutional prioritisation and regulatory support.

In contrast, simulation-based team training and structured debriefing are supported by a more robust body of evidence within the broader healthcare simulation literature. Simulation provides a controlled environment in which communication norms, error management strategies, and feedback practices can be explicitly taught, rehearsed, and refined without risk to patients [21]. Unlike live clinical settings, simulation allows deliberate exposure to critical incidents without patient harm, creating structured opportunities to practice speaking up, challenging authority, and coordinating under stress. Debriefing frameworks, such as advocacy-inquiry, are well described and have demonstrated improvements in team cohesion, perceived psychological safety, and transfer of communication behaviours into clinical environments [22]. However, simulation programs require substantial institutional investment, including faculty training, dedicated space, scheduling flexibility, and financial resources, which may limit scalability in some settings.

Equally important is the redesign of post-operative debriefing and morbidity and mortality conference processes toward a systems-oriented, non-punitive model. Shifting from individual blame to collective analysis of contributing factors promotes error disclosure and reflective practice, both central to adaptive expertise [23]. Structured debriefing approaches have been associated with improved team communication and increased reporting of near-miss events, although variability in implementation quality may influence outcomes. Sustained change requires cultural alignment, senior clinician buy-in, and psychological safety within the very forums intended to promote it--conditions that may take time to establish.

Faculty development remains central to durable cultural transformation. Targeted training in coaching methodologies, emotional intelligence, and bias awareness can shift feedback interactions from evaluative to collaborative. Evidence consistently identifies leader inclusivity and responsiveness to trainee input as strong predictors of psychological safety [24-26]. Nonetheless, faculty development initiatives require ongoing reinforcement, protected time, and accountability mechanisms to prevent regression to entrenched habits, particularly in high-pressure service environments.

The future surgeon: professional identity shaped by culture

The implications of psychological safety extend beyond discrete educational outcomes and into the formation of professional identity within surgical practice. Professional identity formation in medicine is widely understood as a socially constructed process shaped by relational environments, role modelling, and institutional culture rather than technical exposure alone [27]. The future of surgical excellence will therefore be defined not solely by technical precision but by the development of surgeons who consistently demonstrate accountable decision-making, ethical reasoning, and effective collaboration under uncertainty. Psychologically safe training environments support this identity formation process by enabling trainees to integrate technical competence with reflective practice, feedback engagement, and transparent error disclosure, which are behaviours central to professional development [2,24].

Surgeons trained in supportive cultures demonstrate greater openness to feedback, higher tolerance for ambiguity, and stronger engagement in continuous quality improvement processes-behaviours associated with adaptive expertise and improved patient safety outcomes [25]. As surgical practice becomes increasingly interdisciplinary and technology-mediated, competence requires effective communication across professional boundaries, structured error reporting, and adaptive problem-solving in dynamic environments. In this way, psychologically safe environments influence not only trainee experience but also the acquisition of specific skills directly linked to patient safety and team performance.

Ultimately, the surgeon of the future will be shaped as much by the culture in which they train as by the cases they perform. Educational systems that embed psychological safety as a core pedagogical principle will produce clinicians who are not only technically capable but also resilient, reflective, and committed to ethical excellence. In this sense, cultivating safe learning environments represents an investment not merely in trainee wellbeing, but in the long-term integrity and sustainability of the surgical profession itself.
